# Commentary: Selective His-Bundle Pacing May Preserve Intrinsic Repolarization as Well as Depolarization

**DOI:** 10.19102/icrm.2017.080306

**Published:** 2017-03-15

**Authors:** Claudio Tondo


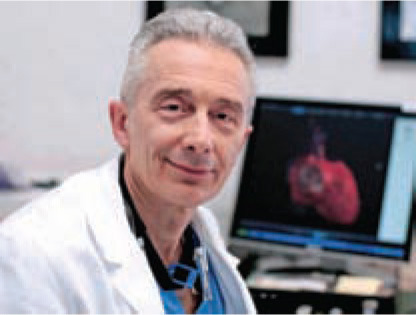


In this issue of the *Journal,* Chinitz et al. introduce an intriguing case of His-bundle pacing as the elected site of pacing in a single chamber pacemaker. It is well-known that His-bundle pacing enhances the preservation of intraventricular depolarization (demonstrated with narrow QRS morphology), and helps to prevent pacing-induced dyssyncrony and potential ventricular dysfunction.

Needless to say, though this is not a new concept, this paper highlights some subtle changes of the ventricular repolarization during high-output His-bundle pacing that promotes earlier ventricular muscle capture (a slurred feature of the initial forces of the QRS) that expresses fusion between the bundle of His and the ventricular muscle. One more time, the precise evaluation of the surface ECG reveals changes due to different modalities of pacing, and not due to other potential causes. The maintenance of physiologic depolarization is therefore of pivotal importance as a means to avoid potential ventricular dysfunction and misinterpretation of repolarization changes. A correct programming of the pacing modality is therefore warranted.

Sincerely,

Claudio Tondo, MD, PhD, FESC

Professor of Cardiology

Chairman, Cardiac Arrhythmia Research Center

Monzino Cardiac Center, IRCCS

Department of Cardiovascular Sciences and Community

University of Milan, Milan, Italy

claudio.tondo@ccfm.it; claudio.tondo@unimi.it

